# Caries Status in People with Dementia: A Systematic Review

**DOI:** 10.3390/jcm14051616

**Published:** 2025-02-27

**Authors:** Weigao Cheng, Dongmin Zhang, Qiwen Li, Han Jiang, Haiying Guo, Minquan Du

**Affiliations:** 1 State Key Laboratory of Oral & Maxillofacial Reconstruction and Regeneration, Key Laboratory of Oral Biomedicine Ministry of Education, Hubei Key Laboratory of Stomatology, School & Hospital of Stomatology, Wuhan University, Wuhan 430072, China; chengweigao@whu.edu.cn (W.C.); zhangdongmin@whu.edu.cn (D.Z.); liqiwen@whu.edu.cn (Q.L.); jianghan@whu.edu.cn (H.J.); 2 Stomatology Center, Zhongshan City People’s Hospital, Zhongshan 528400, China

**Keywords:** oral health, caries, dementia, Alzheimer

## Abstract

**Background and Objective:** People living with dementia typically have poor oral health. However, studies of caries status in this population have revealed different results. This systematic review aimed to assess caries status in old adults with dementia. **Method:** The PubMed, Web of Science, Embase, and Scopus databases were searched from inception to 13 February 2025. The Newcastle–Ottawa Scale (NOS) was used to assess the risk of bias in case–control studies, and the Joanna Briggs Institute (JBI) Critical Appraisal Checklist was used to assess the risk of bias in cross-sectional studies. Caries status was measured by the decayed, missing, filled teeth (DMFT) index, decayed, missing, filled surfaces (DMFS) index, or the component of DMFT/S. A random effects model was used to pool the included data. The weighted mean difference (WMD) and 95% confidence interval (CI) were calculated to analyze the effect of dementia on caries. **Results:** A total of 5363 studies were retrieved, and 20 studies were included in this study. Meta-analysis showed the DMFT index (WMD: 3.76, *p* < 0.0001; 13 studies), decayed teeth (DT) index (WMD: 0.40, *p* < 0.0001; 10 studies), and missing teeth (MT) index (WMD: 3.67, *p* = 0.04; 7 studies) values were higher in the dementia group than the control group. There were no differences in the filled teeth (FT) index (WMD: −0.66, *p* = 0.09; 9 studies) between the dementia group and the control group. **Conclusions:** Caries status was poorer in people with dementia than the controls. These findings suggest that medical staff and caregivers need to pay more attention to the oral health of dementia patients.

## 1. Introduction

Dementia is a syndrome accompanied by a deterioration in cognitive performance and impairments in functional ability [1]. This syndrome is not a singular disease but rather an umbrella term encompassing various conditions, including Alzheimer’s disease (AD), vascular dementia (VD), and frontotemporal dementia [2]. People with dementia often lose the functional ability to care for themselves and exhibit poor compliance with caregivers [3], which makes them susceptible to infections. Current clinical strategies include pharmacologic interventions for cognitive maintenance and behavioral regulation, as well as multimodal psychosocial approaches for functional preservation. However, there is no disease-modifying treatments to halt or reverse underlying neurodegeneration [4]. According to the World Health Organization (WHO), the number of people with dementia around the world was more than 55 million in 2019 and will rise to 139 million in 2050 [5], thereby increasing the economic and health care burdens on societies and families.

The oral cavity is the second most important bacterial habitat within the human body. It is well known that people with dementia often face challenges in maintaining their oral hygiene [6]. In addition, people with dementia often experience a reduction in saliva production, which may be related to the condition itself as well as the medications taken for dementia [7]. These factors lead to the gradual accumulation of food debris and the proliferation of bacteria in the oral cavity. Therefore, people with dementia may be susceptible to caries, a common oral infection characterized by progressive destruction of the hard tissue of the teeth [8]. Caries can cause pain, diminish masticatory performance, and have a further negative impact on nutrition and overall quality of life [8]. Thus, it is important to gain a clear understanding of caries status among people with dementia.

However, clinical studies examining the impact of dementia on caries yielded inconsistent results. Some studies reported that people in the dementia group had higher decayed, missing, filled teeth/surfaces (DMFT/DMFS) values than people with normal cognitive function [9,10,11], while other studies showed no difference between these two groups [12,13]. Therefore, we performed the current systematic review to comprehensively assess the caries status of dementia patients. Our objective was to establish a foundation for targeted dental care strategies and policies aimed at providing early intervention and enhancing oral health outcomes for people with dementia.

## 2. Materials and Methods

### 2.1. Search Strategy

The PubMed, Web of Science, Embase, and Scopus databases were searched from inception to 13 February 2025. The following keywords related to caries were used for the search: caries, decayed teeth, missing teeth, filled teeth, oral health, dental health, and oral hygiene. The following keywords related to dementia were used for the search: dementia, cognitive impairment, cognitive decline, and Alzheimer. The search strategies were tailored for each database. The detailed search strategies were shown in the Supplementary Material (Supplementary File S1).

### 2.2. Inclusion and Exclusion Criteria

We retrieved observational studies which examined the association between caries and dementia. We included studies which measured caries status using the DMFT index, the DMFS index, or the component of DMFT/S. The exclusion criteria were as follows: (1) DMFT/S indices or their component not be extracted; (2) studies without a control group; (3) data of DMFT/S presented as medians; (4) studies that only exanimated half-mouth; (5) case report, meeting abstract and review; (6) duplicate studies; and (7) studies which were not published in English.

### 2.3. Study Screening and Data Extraction

Two researchers independently screened the studies based on the inclusion and exclusion criteria, and extracted the data of the included studies. The following data were extracted: first author, year of publication, study design, mean age, diagnostic criteria for dementia, and sample size. The mean and standard deviation (SD) values of the caries indices, and the mean deviation (MD) between the dementia group and the control group were also extracted. For longitudinal studies, only baseline data were extracted. Disagreements were settled through discussion or by consulting a third researcher.

### 2.4. Quality Assessment

The quality assessment was independently conducted by two researchers. All longitudinal studies were evaluated as case–control studies on the basis of the baseline trial design. The Newcastle–Ottawa Scale (NOS) was used to assess the quality of the case–control studies [14]. The total score of the NOS was 9. Scores of 0–3, 4–6, and 7–9 corresponded to low, moderate, and high quality, respectively. The quality of the cross-sectional studies was evaluated by the Joanna Briggs Institute (JBI) Critical Appraisal Checklist for Analytical Cross-Sectional Studies. The checklist consists of 8 items answered yes, no, unclear, or not applicable. These results were classified into “Include”, “Exclude”, and “Seek further info” [15].

### 2.5. Statistical Analysis

The weighted mean difference (WMD) and its 95% confidence interval (CI) were used to investigate the difference in caries status between dementia patients and the controls. I^2^ was used to measure the heterogeneity among studies. The value exceeding 50% indicated high heterogeneity. Sensitivity analysis was conducted by excluding one study in turn. Subgroup analysis was conducted to explore heterogeneity across studies, according to type of dementia, mean age of dementia patient, year of survey, and study design. *p* value less than 0.05 were considered to statistical significance. RevMan 5.4 software was used to perform the analysis.

## 3. Results

### 3.1. Included Studies

**Figure 1:**
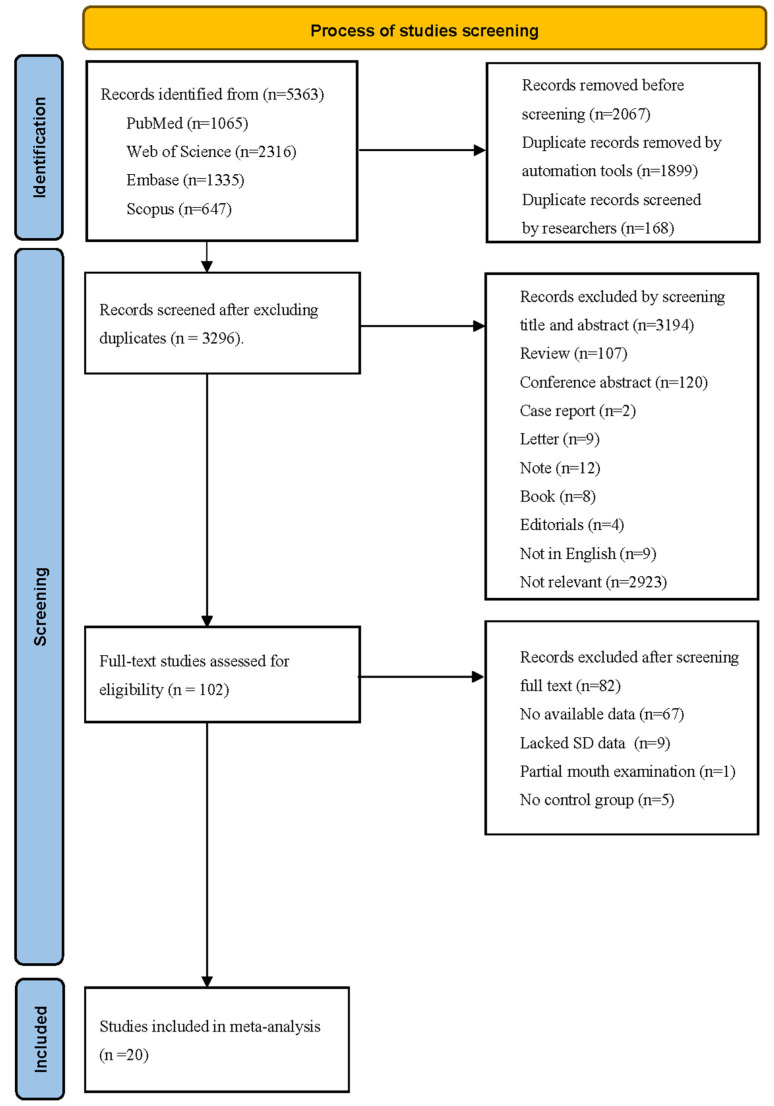
PRISMA flow diagram for research studies included in this systematic review.

A total of 5363 studies were retrieved from three databases. After removing duplicates, 3296 studies remained. Upon screening the titles and abstracts, 3194 studies were excluded for various reasons, such as being review, conference abstract, case report, letter, note, book, editorials, non-English article, or being unrelated to the effect of dementia on dental caries. Of the remaining 102 studies for full-text reading, 67 studies lacked DMFT/S indices, 9 studies lacked SD data [16,17,18,19,20,21,22,23,24], 1 study only described partial mouth examinations [25], and 5 studies lacked a control group [26,27,28,29,30]. Ultimately, 20 studies were included in the systematic review, comprising 13 cross-sectional studies [10,12,13,24,31,32,33,34,35,36,37,38,39] and 7 case–control studies [9,11,40,41,42,43,44]. The screening process was outlined in Figure 1.

### 3.2. Main Characteristics of the Included Studies

The characteristics of the included studies were shown in Table 1. The sample sizes of these studies ranged from 40 to 1797. The measurements and diagnostic criteria for dementia varied across these included studies. For example, the Mini-Mental Status Examination (MMSE) [13,31,41] and the Clinical Dementia Rating (CDR) scores [31,33] were commonly used to assess dementia. The International Classification of Diseases, Tenth Revision Criteria (ICD-10) [31,34], and Diagnostic and Statistical Manual of Mental Disorders-IV (DSM-IV) criteria [11,31] were frequently used for dementia diagnosis. Additionally, four studies identified dementia cases via medical records [32,36,37,38]. Among the included studies, nine studies enrolled participants without dementia as the control group [12,24,33,34,35,36,37,38,39], while another nine studies included healthy individuals as the control group [9,11,13,32,40,41,42,43,44], and two studies did not clearly indicate the participants’ cognition in control group [10,31].

As shown in Table 2, the findings concerning the caries status of people living with dementia were inconsistent. The dementia group exhibited a higher DMFT index value than the control group in six studies [9,10,11,35,40,41], whereas five studies reported no difference in this index between the two groups [13,36,37,38,43]. Three studies reported a greater value of the DT index in the dementia group than that in the control group [24,33,39], whereas no difference was found in six studies [11,12,35,38,40,43]. Two studies concluded that people with dementia were more likely to miss teeth than the controls were [11,40], whereas four studies reported no difference in the MT index between the two groups [12,35,38,43]. In terms of the FT index, two studies reported lower values in the dementia group [33,40], but the difference was not significant in six studies [11,12,32,35,38,43].

For coronal caries, no differences were found in the coronal decayed teeth (CDT) index [9,36,44], coronal filled teeth (CFT) index [9,36], coronal decayed surfaces (CDS) index [34,44], or coronal decayed and filled surfaces (CDFS) [34,44] index between the dementia and control groups. The conclusions of the root caries indices were inconsistent. Ellefsen et al. reported a significantly greater value of the root decayed surfaces (RDS) index in the dementia group, and no difference in the root decayed and filled surfaces (RDFS) index [34]. Conversely, Jones et al. reported that there was no difference in the RDS index, and the RDFS index value in the dementia group was lower than that in the control group [44].

### 3.3. Quality Assessment of the Included Studies

**Figure 2:**
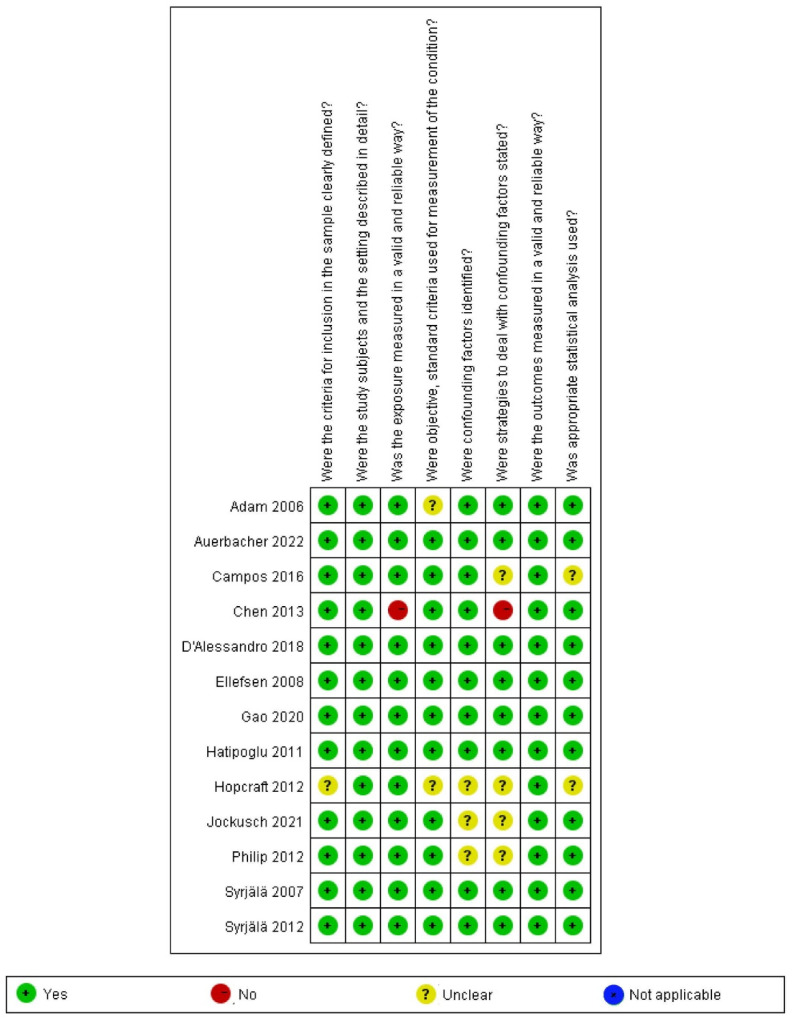
Risk of bias analysis of cross-sectional studies [10,12,13,24,31,33,34,35,36,37,38,39,40].

**Figure 3:**
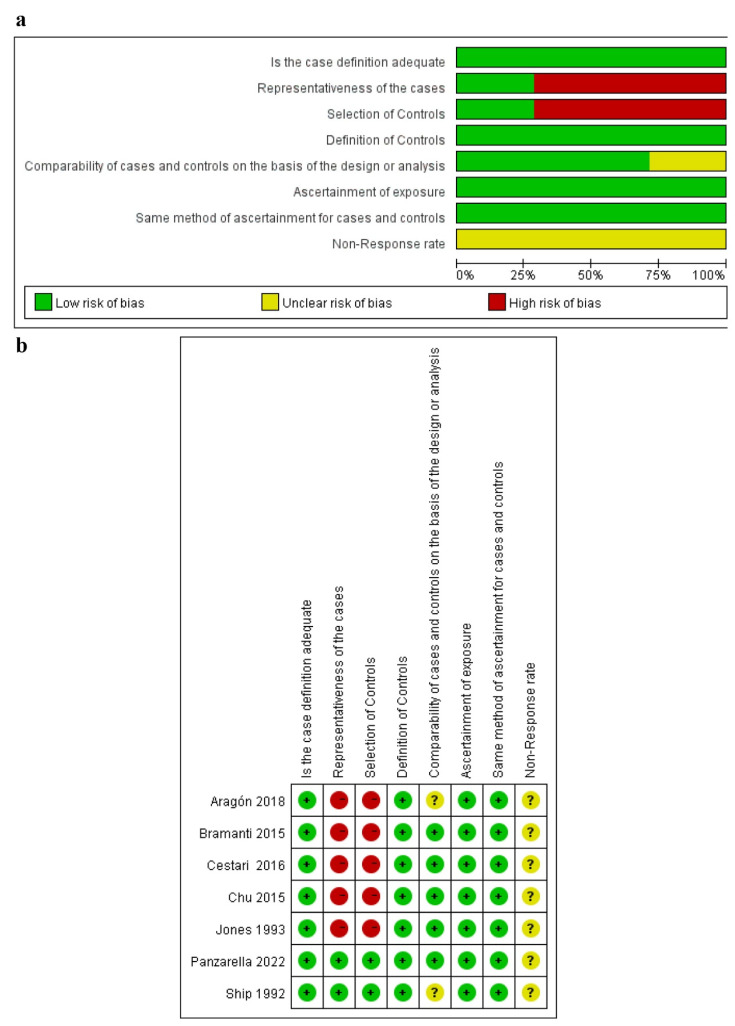
Risk of bias analysis of case–control studies [9,11,32,41,42,43,44]. (**a**) Summary of bias risks across case-control studies; (**b**) Individual study-level bias assessment, where green/yellow/red indicate low/some concerns/high risk of bias respectively.

According to the JBI Critical Appraisal Checklist for Analytical Cross-Sectional Studies, twelve cross-sectional studies obtained more than five “yes” responses in the article quality assessment [10,12,13,24,31,32,33,34,35,37,38,39], and one study obtained only three “yes” responses [36]; thus, those were evaluated as “include” (as shown in Figure 2; details shown in Supplementary Table S1). Among the seven case–control studies, six studies were evaluated as “moderate” quality [9,40,41,42,43,44], and one study was rated as “high” quality [11] (as shown in Figure 3; details shown in Supplementary Table S1).

### 3.4. Meta-Analysis

#### 3.4.1. DMFT Index

**Figure 4:**
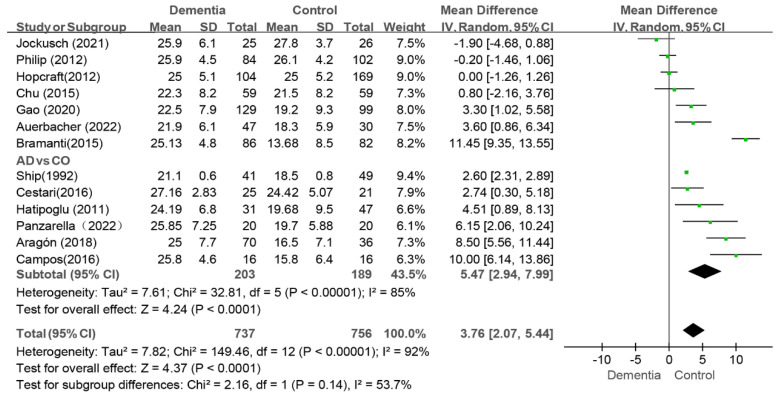
Meta-analysis results of DMFT index of dementia patients in comparison to controls [9,10,11,13,31,32,35,37,38,39,41,42,43]. Green squares: the mean differences of individual study; Black diamond: the pooled weighted mean difference from meta-analysis.

A meta-analysis of 13 studies revealed that people with dementia had a higher DMFT index than the controls (WMD: 3.76, 95%CI (2.07, 5.44), *p* < 0.0001, I^2^ = 92%) [9,10,11,13,31,35,36,37,38,40,41,42,43]. The I^2^ value was greater than 75% even after the sequential exclusion of studies, and the results were consistent. These findings indicated that dementia patients had higher DMFT scores than the controls (as shown in Figure 4).

Among these studies, six specifically evaluated the caries status of AD patients [9,11,13,31,40,42]. In this subgroup, the value of the DMFT index in the AD group was greater than that in the control group (WMD: 5.47, 95%CI (2.94, 7.99), *p* < 0.0001, I^2^ = 85%) (as shown in Figure 4). After two studies were removed, the I^2^ decreased to 41% [9,42]. The sensitivity analysis revealed that the results concerning the effect of AD on caries were stable.

#### 3.4.2. DT Index

**Figure 5:**
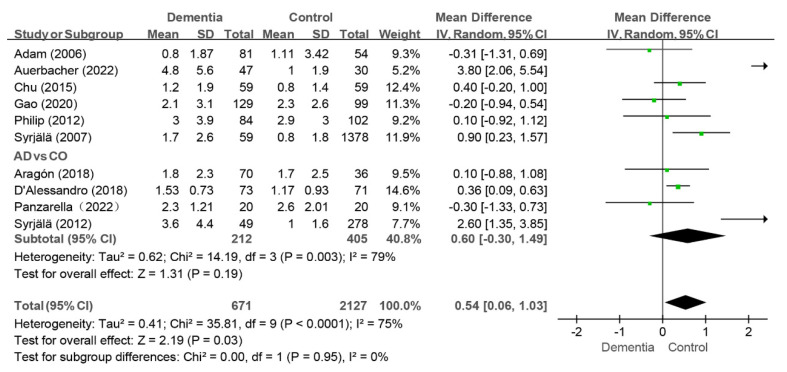
Meta-analysis results of DT index of dementia patients in comparison to controls [10,11,12,24,33,35,39,40,41,43]. Green squares: the mean differences of individual study; Black diamond: the pooled weighted mean difference from meta-analysis.

Ten studies reported the DT index values, and the pooled analysis revealed that the dementia group had higher scores than the control group (WMD: 0.54, 95%CI (0.06, 1.03), *p* = 0.03, I^2^ = 75%) [10,11,12,24,33,35,38,39,40,43] (as shown in Figure 5). The I^2^ value remained above 50% after individual studies were removed in the sensitivity analysis, and the findings remained consistent. Moreover, when the two studies with MD of 3.80 and 2.60, respectively, were removed together in the sensitivity analysis, the I^2^ value dropped to 14%, while the results still remained consistent [10,24]. When AD patients were compared with controls in four studies, no difference was observed between two group (WMD: 0.60, 95%CI (−0.30, 1.49), *p* = 0.19, I^2^ = 79%) [11,24,33,40] (as shown in Figure 5). Further sensitivity analysis revealed that the difference in DT status between the AD group and the control group were consistent.

#### 3.4.3. MT Index

**Figure 6:**
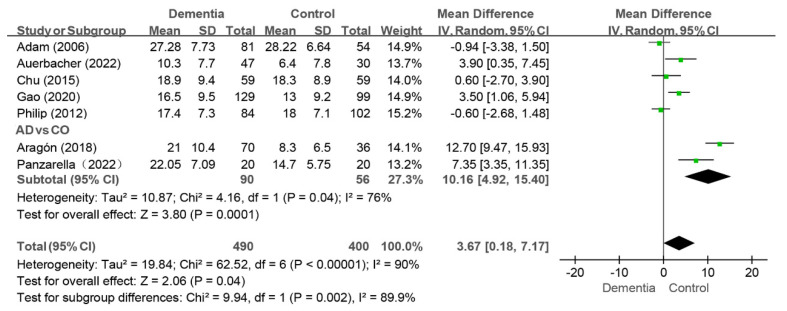
Meta-analysis results of MT index of dementia patients in comparison to controls [10,11,12,35,39,41,43]. Green squares: the mean differences of individual study; Black diamond: the pooled weighted mean difference from meta-analysis.

Seven studies reported the MT index, and the pooled analysis revealed that the score was higher in the dementia group than that in the control group (WMD: 3.67, 95%CI (0.18, 7.17), *p* = 0.04, I^2^ = 90%) [10,11,12,35,38,40,43]. Notably, no difference in the MT index between groups was observed when four studies were removed individually [10,11,35,40]. Two studies compared the MT index of the AD group to that of the control group [11,40], and the MT value was greater in the AD group than that in the control group (WMD: 10.16, 95%CI (4.92, 15.40), *p* = 0.0001, I^2^ = 76%) (as shown in Figure 6).

#### 3.4.4. FT Index

**Figure 7:**
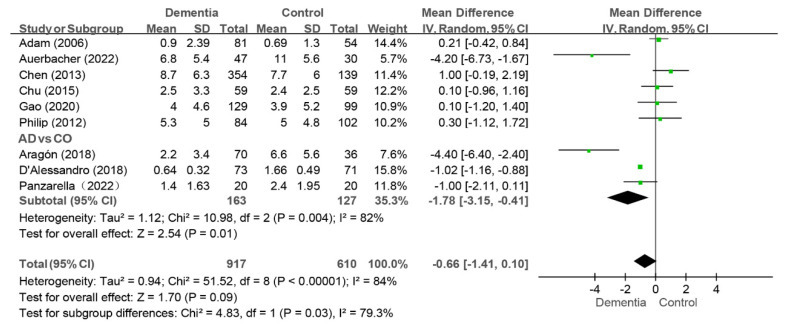
Meta-analysis results of FT index of dementia patients in comparison to controls [11,33,40]. Green squares: the mean differences of individual study; Black diamond: the pooled weighted mean difference from meta-analysis.

Nine studies reported the FT index, and the pooled analysis revealed that there were no difference between the dementia group and the control group (WMD: −0.66, 95%CI (−1.41, 0.10), *p* = 0.09, I^2^ = 84%) [10,11,12,32,33,35,38,40,43] (as shown in Figure 7). Sensitivity analysis revealed that the value of the FT index in the dementia group was lower than that in the control group (WMD: −0.85, 95%CI (−1.62, −0.08), *p* = 0.03, I^2^ = 83%) after the study by Chen et al. was removed [32], indicating instability in the results.

Among the nine studies, three compared the FT index of the AD group with those of the control group [11,33,40], and the AD group presented a lower FT index than the control group did (WMD: −1.78, 95%CI (−3.15, −0.41), *p* = 0.01, I^2^ = 82%) (as shown in Figure 7). The conclusion was consistent in the sensitivity analysis.

#### 3.4.5. CDT, CFT, CDS, CDFS, RDS and RDFS Indexes

**Figure 8:**
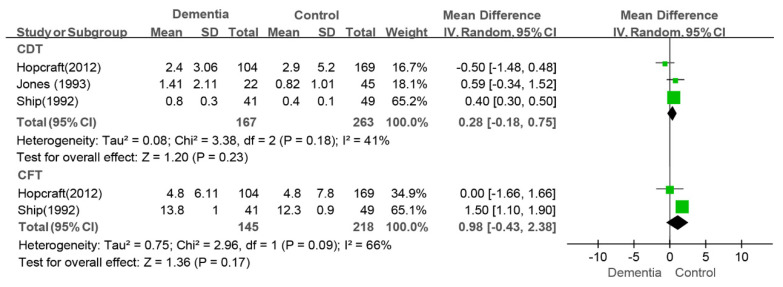
Meta-analysis results of CDT [9,38,44] and CFT [9,38] indices of dementia patients in comparison to controls. Green squares: the mean differences of individual study; Black diamond: the pooled weighted mean difference from meta-analysis.

The meta-analysis of three studies assessing the CDT index did not reveal a difference between the dementia group and the control group (WMD: 0.28, 95%CI (−0.18, 0.75), *p* = 0.18, I^2^ = 41%) [9,36,44] (as shown in Figure 8). The conclusion was inconsistent in the sensitivity analysis. That is, a greater value was observed in the dementia group than in the control group (WMD: 0.40, 95%CI (0.31, 0.50), *p* < 0.00001, I^2^ = 0%) after the study by Hopcraft et al. was removed [36]. There was also no difference in the CFT index (WMD: 0.98, 95%CI (−0.43, 2.38), *p* = 0.69, I^2^ = 0%) between the dementia group and the control group (as shown in Figure 8).

**Figure 9:**
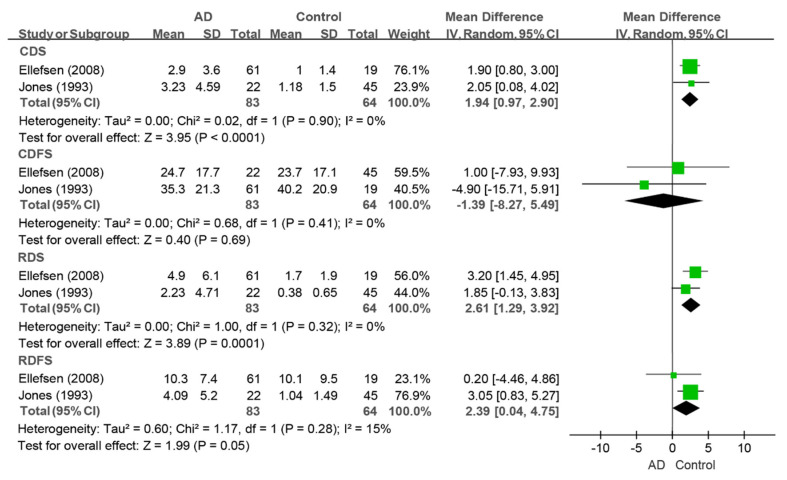
Meta-analysis results of the CDS, CDFS, RDS, and RDFS indices of dementia patients in comparison to controls [34,44]. Green squares: the mean differences of individual study; Black diamond: the pooled weighted mean difference from meta-analysis.

Only two studies that evaluated the CDS, CDFS, RDS, and RDFS indices could be pooled for meta-analysis [34,44]. People living with dementia had greater values of the CDS index (WMD: 1.94, 95%CI (0.97, 2.90), *p* < 0.0001, I^2^ = 0%), RDS index (WMD: 2.61, 95%CI (1.29, 3.92), *p* = 0.0001, I^2^ = 0%), and RDFS index (WMD: 2.39, 95%CI (0.04, 4.75), *p* = 0.05, I^2^ = 15%) than the controls did. There was no difference in the CDFS index (WMD: −1.39, 95%CI (−8.27, 5.49), *p* = 0.69, I^2^ = 0%) between the two groups (as shown in Figure 9).

### 3.5. Subgroup Analysis

Subgroup analysis revealed that a higher MT index was associated with the type of dementia (AD) and year of survey (>2015). A lower FT index was associated with the type of dementia (AD), mean age of dementia (<80), and year of survey (>2015). Details are shown in Table 3.

## 4. Discussion

The aim of this study was to compare the caries status between people with dementia and people without dementia. The result revealed that the caries status was worse in people living with dementia than the controls, and that dementia patients had fewer filled teeth than the controls did.

Dental caries is a common oral disease that is caused by the oral biofilm flora [8,46]. Inadequate oral hygiene practices can lead to the accumulation of food residues and oral bacteria, increasing the risk of caries. Many studies have demonstrated that people with dementia have poor oral hygiene [47,48,49,50,51,52]. Several factors contribute to poor oral hygiene in dementia patients. First, the cognitive decline associated with dementia leads to a progressive decline in the ability to perform self-oral care and seek medical attention [53,54,55]. Furthermore, individuals with dementia can exhibit aggressive behavior towards oral care providers [3,56]. Finally, oral health is often overlooked in people living with dementia [57,58]. Both people with dementia and their caregivers lack an understanding of the importance of oral hygiene, which leads to a failure to perform oral hygiene and further results in poor oral health in dementia patients [59,60]. In a word, dementia patients cannot maintain good oral hygiene, which could lead to more dental caries.

Despite poor oral hygiene, the low flow rate and weak buffering capacity of saliva can also increase the incidence of dental caries [61]. People with dementia have lower salivary flow rates than individuals without dementia [41,43]. Additionally, dementia patients have lower salivary pH values and a weaker buffering capacity than the controls do [41]. In addition, some medicines prescribed for people with dementia, such as antipsychotic medications and cholinesterase inhibitors, have side effects on the salivary flow rate and buffering capacity [62,63,64]. In short, poor oral hygiene and decreases in saliva buffering capacity and flow rate increase the susceptibility of dementia patients to caries. Thus, the finding in this meta-analysis that dementia patients have a higher DMFT index and DT index than the controls is well-founded.

Tooth filling is a crucial procedure for restoring the shape and function of teeth, and requires active cooperation from the patient. Thus, it is difficult for people living with dementia to cooperate with dentists [41,65]. In addition, timely treatment of caries is often delayed because people with dementia and their caregivers often neglect oral health. Consequently, it is likely that people with dementia have lower values of the FT/CFT index than controls do.

This study systematically compared the caries status of elderly individuals with dementia to that of the control group. It is beneficial for the general public to be aware of the prevalence of caries and to pay attention to the oral health of the dementia population. However, there are several limitations to this study. First, the types of dementia were not clearly specified in these included studies. Some studies included both AD patients and other types of dementia patients in the dementia group [24], whereas other studies recruited only AD patients [13]. Notably, people with VD often exhibit weakness or impaired motor function [66], which can lead to distinct effects on oral health. Second, the severity of dementia was not explicitly addressed in these included studies. The varying degrees of dementia severity may impact the ability to perform oral hygiene practices, thereby influencing oral health status. Specifically, patients with moderate and severe dementia may refuse to receive oral health interventions, and/or show limited cooperation, whereas those with mild dementia may exhibit better cooperation [47,48]. Third, the DMFT score and related indicators have some limitations in accurately assessing caries status. DMFT and other indicators focus only on the number of caries but cannot reflect information on active dental caries or the severity of dental caries [67,68]. Owing to the lack of attention given to non-cavitated lesions, DMFT and other indicators cannot provide accurate data about lesions at early stages [67,69], which may lead to an underestimation of caries. Conversely, the severity of the caries status may be exaggerated because the MT index could be overestimated if teeth are extracted for reasons other than caries, such as trauma, orthodontic needs, or periodontal disease [68]. Finally, owing to language restrictions and the lack of grey literature retrieval, the number of included studies may have been reduced. Additionally, because of the limitation of analyzable data, we were unable to assess the impact of potential confounding variables, such as sex, socioeconomic status, and access to dental care. These limitations should be taken into account when the findings are interpreted, and future research efforts should aim to address these gaps to enhance our understanding of the relationship between dementia and caries.

## 5. Conclusions

In conclusion, the results of this systematic review clearly showed that caries status was worse in people with dementia than in people without dementia. Notably, several studies explicitly reported significantly worse caries outcomes in dementia people [9,10,11], underscoring the urgent need for targeted interventions. To improve the oral health status of patients with dementia, it is imperative to raise awareness about the importance of oral care among the public and caregivers, to increase nursing literacy among caregivers, and to enhance oral health guidance and support for patients with dementia. In addition, more high-quality studies are needed to clarify the relationship between dementia and caries status, and to deliver more specific guidance for clinical practice.

## Figures and Tables

**Table 1:** Main characteristics of datasets from the included studies.

Studies	Location	Study Design	Mean Age	Mean Age	Dementia	Caries Indexes
First Author (Year)			(Dementia)	(Control)	Measurement	
Adam (2006) [12]	UK	cross- sectional	80.78	85.48	AMT	DT/MT/FT
Aragón (2018) [41]	German	case–control	77.4	62.6	McKhann et al. [45] diagnosed criteria	DMFT/DT/MT/FT
Auerbacher (2022) [10]	German	cross- sectional	76.7	61.5	medical records	DMFT/DT/MT/FT
Bramanti (2015) [32]	Italy	case–control	82.7	80.2	MMSE	DMFT
Campos (2016) [31]	Brazil	cross- sectional	76.7	51.7	ICD-10	DMFT
Cestari (2016) [42]	Brazil	case–control	77.68	75.33	NINCDS-ADRDA	DMFT
Chen (2013) [36]	USA	cross- sectional	82.6	76.1	medical records	FT
Chu (2015) [43]	China	case–control	79.8	unclear	unclear	DMFT/DT/MT/FT
D’Alessandro (2018) [33]	Italy	cross- sectional	79.1	77.68	CDR	DT/FT
Ellefsen (2008) [34]	Denmark	cross- sectional	82.4	79.8	ICD-10	CDS/CDFS/RDS/RDFS
Gao (2020) [39]	China	cross- sectional	80.9	79.4	unclear	DMFT/DT/MT/FT
Hatipoglu (2011) [13]	Turkey	cross- sectional	67.61	65.32	MMSE	DMFT
Hopcraft (2012) [38]	Australia	cross- sectional	unclear	unclear	medical records	DMFT/CDT/CMT/CFT
Jockusch (2021) [37]	Switzerland	cross- sectional	87	75	MMSE	DMFT
Jones (1993) [44]	USA	case–control	67.4	66.1	unclear	CDT/CDS/CDFS/RDS/RDFS
Panzarella (2022) [11]	Italy	case–control	83.5	78.8	DSM-IV-TR	DMFT/DT/MT/FT
Philip (2012) [35]	Australia	cross- sectional	85.7	84.3	medical records	DMFT/DT/MT/FT
Ship (1992) [9]	USA	case–control	68.2	64.1	NINCDS-ADRDA	DMFT/CDT/CFT
Syrjälä (2007) [40]	Finland	cross- sectional	76.3	66.4	shorten MMSE	DT
Syrjälä (2012) [24]	Finland	cross- sectional	84.8	81.4	DSM-IV	DT

Abbreviations: AMT, Abbreviated mental test; CDR, the Clinical Dementia Rating score; CDT, coronal decayed teeth; CDS, coronal decayed surfaces; CDFS, coronal decayed and filled surfaces; CFT, coronal filled teeth; DMFT, Decayed (D), Missing (M), Filled (F) Teeth; DSM-IV, Diagnostic and statistical manual of mental disorders, fourth edition; DSM-IV-TR, Diagnostic and statistical manual of mental disorders, fourth edition, text revision; ICD-10, International Classification of Diseases, Tenth Revision; MMSE, Mini-mental State Examination; NINCDS-ADRDA, National Institute of Neurological and Communicative Disorders and Stroke and the Alzheimer’s Disease and Related Disorders Association; RDS, root decayed surfaces; RDFS, root decayed and filled surfaces; shorten MMSE, a shortened version of the Mini Mental State Examination.

**Table 2:** Caries data extracted from the included studies.

Study	Dementia		Control			*p* Value
	Mean ± SD	N	Mean ± SD	N	MD	
**DMFT**						
Auerbacher (2022) [10]	21.9 ± 6.1	47	18.3 ± 5.9	30	3.6	**<0.05**
Aragón (2018) [41]	25 ± 7.7	70	16.5 ± 7.1	36	8.5	**<0.001**
Bramanti (2015) [32]	25.13 ± 4.8	86	13.68 ± 8.5	82	11.45	**<0.05**
Campos (2016) [31]	25.8 ± 4.6	16	15.8 ± 6.4	16	10	unclear
Cestari (2016) [42]	27.16 ± 5.83	25	24.42 ± 5.07	21	2.74	unclear
Chu (2015) [43]	22.3 ± 8.2	59	21.5 ± 8.2	59	0.8	0.59
Gao (2020) [39]	22.5 ± 7.9	129	19.2 ± 9.3	99	3.3	**0.041**
Hatipoglu (2011) [13]	24.19 ± 6.8	31	19.68 ± 9.50	47	4.51	0.126
Hopcraft (2012) [38]	25 ± 5.1	104	25 ± 5.2	169	0	>0.05
Jockusch (2021) [37]	25.9 ± 6.1	25	27.8 ± 3.7	26	−1.9	>0.05
Panzarella (2022) [11]	25.85 ± 7.25	20	19.70 ± 5.88	20	5.88	**0.001**
Philip (2012) [35]	25.9 ± 4.5	84	26.1 ± 4.2	102	−0.2	>0.05
Ship (1992) [9]	21.1 ± 0.6	41	18.5 ± 0.8	49	2.6	**0.02**
**DT**						
Adam (2006) [12]	0.80 ± 1.87	81	1.11 ± 3.42	54	−0.31	>0.05
Aragón (2018) [41]	1.8 ± 2.3	70	1.7 ± 2.5	36	0.1	0.77
Auerbacher (2022) [10]	4.8 ± 5.6	47	1.0 ± 1.9	30	3.8	unclear
Chu (2015) [43]	1.2 ± 1.9	59	0.8 ± 1.4	59	0.4	0.28
D’Alessandro (2018) [33]	1.53 ± 0.73	73	1.17 ± 0.93	71	0.36	**0.005**
Gao (2020) [39]	2.1 ± 3.1	129	2.3 ± 2.6	99	−0.2	0.997
Panzarella (2022) [11]	2.3 ± 1.21	20	2.60 ± 2.01	20	−0.3	>0.05
Philip (2012) [35]	3 ± 3.9	84	2.9 ± 3.0	102	0.1	>0.05
Syrjälä (2007) [40]	1.7 ± 2.6	59	0.8 ± 1.8	1378	0.9	**0.04**
Syrjälä (2012) [24]	3.6 ± 4.4	49	1.0 ± 1.6	278	2.6	unclear
**MT**						
Adam (2006) [12]	27.28 ± 7.73	81	28.22 ± 6.64	54	−0.94	>0.05
Aragón (2018) [41]	21.0 ± 10.4	70	8.3 ± 6.5	36	12.7	**<0.001**
Auerbacher (2022) [10]	10.3 ± 7.7	47	6.4 ± 7.8	30	3.9	unclear
Chu (2015) [43]	18.9 ± 9.4	59	18.3 ± 8.9	59	0.6	0.75
Gao (2020) [39]	16.5 ± 9.5	129	13.0 ± 9.2	99	3.5	0.528
Panzarella (2022) [11]	22.05 ± 7.09	20	14.70 ± 5.75	20	7.35	**<0.001**
Philip (2012) [35]	17.4 ± 7.3	84	18.0 ± 7.1	102	−0.6	>0.05
**FT**						
Adam (2006) [12]	0.9 ± 2.39	81	0.69 ± 1.30	54	0.21	>0.05
Aragón (2018) [41]	2.2 ± 3.4	70	6.6 ± 5.6	36	−4.4	**<0.001**
Auerbacher (2022) [10]	6.8 ± 5.4	47	11.0 ± 5.6	30	−4.2	unclear
Chen (2013) [36]	8.7 ± 6.3	354	7.7 ± 6.0	139	1	>0.05
Chu (2015) [43]	2.5 ± 3.3	59	2.4 ± 2.5	59	0.1	0.88
D’Alessandro (2018) [33]	0.64 ± 0.32	73	1.66 ± 0.49	71	−1.02	**<0.001**
Gao (2020) [39]	4 ± 4.6	129	3.9 ± 5.2	99	0.1	0.677
Panzarella (2022) [11]	1.40 ± 1.63	20	2.40 ± 1.95	20	−1	>0.05
Philip (2012) [35]	5.3 ± 5.0	84	5.0 ± 4.8	102	0.3	>0.05
**CDT**						
Jones (1993) [44]	1.41 ± 2.11	22	0.82 ± 1.01	45	0.59	0.23
Hopcraft (2012) [38]	2.4 ± 3.06	104	2.9 ± 5.2	169	−0.5	>0.05
Ship (1992) [9]	0.8 ± 0.3	41	0.4 ± 0.1	49	0.4	>0.05
**CDS**						
Ellefsen (2008) [34]	2.9 ± 3.9	87	1.0 ± 1.4	19	1.9	>0.05
Jones (1993) [44]	3.23 ± 4.59	22	1.18 ± 1.50	45	2.05	**0.053**
**CDFS**						
Ellefsen (2008) [34]	35.3 ± 22.0	87	40.2 ± 20.9	19	−4.9	>0.05
Jones (1993) [44]	24.7 ± 17.7	22	23.7 ± 17.1	45	1	0.83
**RDS**						
Ellefsen (2008) [34]	4.1 ± 5.6	87	1.7 ± 1.9	19	2.4	>0.05
Jones (1993) [44]	2.23 ± 4.71	22	0.38 ± 0.65	45	1.85	0.081
**RDFS**						
Ellefsen (2008) [34]	8.9 ± 7.3	87	10.1 ± 9.5	19	−1.2	>0.05
Jones (1993) [44]	4.09 ± 5.20	22	1.04 ± 1.49	45	3.05	**0.013**
**CFT**						
Hopcraft (2012) [38]	4.8 ± 6.11	104	4.8 ± 7.8	169	0	>0.05
Ship (1992) [9]	13.8 ± 1.0	41	12.3 ± 0.9	49	1.5	>0.05

Abbreviations: DMFT, Decayed (D), Missing (M), Filled (F) Teeth; CDT, coronal decayed teeth; CDS, coronal decayed surfaces; CFT, coronal filled teeth; CDFS, coronal decayed and filled surfaces; RDS, root decayed surfaces; RDFS, root decayed and filled surfaces.

**Table 3:** Subgroup analyses between the dementia and control group.

Subgroup	Categories	Studies	Sample Size		Weight	MD [95%CI]	I^2^ (%)	*p* Value	*p* Value Within Subgroup
			Dementia	Control					
**(1) Subgroup analyses of the DMFT index**									
Type of dementia	Other dementia	7	534	567	56.50%	2.44 [−0.71, 5.59]	94	0.13	0.14
	Alzheimer’s disease	6	203	189	43.50%	5.47 [2.94, 7.99]	85	<0.0001	
Mean age of dementia patient	>80	4	319	303	37.30%	5.13 [−0.70, 10.96]	97	0.08	0.82
	<80	7	289	258	62.70%	4.40 [2.41, 6.38]	81	<0.0001	
Year of survey	≤2015	6	405	508	49.40%	3.09 [0.61, 5.57]	95	0.01	0.82
	>2015	7	332	248	50.60%	4.47 [1.71, 7.23]	84	0.002	
Study design	cross-sectional	7	425	462	53.60%	2.56 [0.32, 4.80]	86	0.02	0.18
	case–control	6	301	267	46.40%	5.34 [1.98, 8.71]	94	0.002	
**(2) Subgroup analyses of the DT index**									
Type of dementia	Other dementia	6	459	1722	36.30%	0.42 [0.08, 0.75]	77	0.01	0.92
	Alzheimer’s disease	4	212	405	63.70%	0.39 [0.14, 0.64]	79	0.002	
Mean age of dementia patient	>80	5	363	553	46.50%	0.31 [−0.60, 1.23]	77	0.5	0.44
	<80	5	308	1574	53.50%	0.75 [0.13, 1.37]	76	0.02	
Year of survey	≤2015	5	332	1871	50.40%	0.67 [−0.07, 1.42]	74	0.08	0.7
	>2015	5	339	256	49.60%	0.46 [−0.32, 1.24]	79	0.25	
Study design	cross-sectional	7	522	2012	69.10%	0.80 [0.11, 1.50]	82	0.02	0.15
	case–control	3	149	115	30.90%	0.19 [−0.26, 0.65]	0	0.41	
**(3) Subgroup analyses of the MT index**									
Type of dementia	Other dementia	5	400	344	72.70%	1.14 [−0.88, 3.15]	65	0.27	**0.002**
	Alzheimer’s disease	2	90	56	27.30%	10.16 [4.92, 15.40]	76	0.0001	
Mean age of dementia patient	>80	4	314	275	58.10%	2.03 [−1.20, 5.27]	84	0.22	0.36
	<80	3	176	125	41.90%	5.75 [−1.53, 13.02]	93	0.12	
Year of survey	≤2015	3	224	215	44.00%	−0.49 [−1.92, 0.94]	0	0.5	**0.002**
	>2015	4	266	185	56.00%	6.82 [2.42, 11.23]	86	0.002	
Study design	cross-sectional	4	341	285	58.60%	1.29 [−1.19, 3.77]	73	0.31	0.16
	case–control	3	149	115	41.40%	6.88 [−0.45, 14.21]	92	0.07	
**(4) Subgroup analyses of the FT index**									
Type of dementia	Other dementia	6	754	483	64.70%	−0.00 [−0.80, 0.79]	63	1	**0.03**
	Alzheimer’s disease	3	163	127	35.30%	−1.78 [−3.15, −0.41]	82	0.01	
Mean age of dementia patient	>80	5	668	414	58.70%	0.11 [−0.48, 0.70]	35	0.71	**0.01**
	<80	4	249	196	41.30%	−1.97 [−3.51, −0.43]	86	0.01	
Year of survey	≤2015	4	578	354	48.20%	0.32 [−0.15, 0.78]	0	0.18	**0.001**
	>2015	5	339	256	51.80%	−1.68 [−2.82, −0.54]	80	0.004	
Study design	cross-sectional	6	768	495	68.40%	−0.32 [−1.24, 0.60]	86	0.5	0.28
	case–control	3	149	115	31.60%	−1.59 [−3.70, 0.51]	87	0.14	

Abbreviations: DMFT, Decayed (D), Missing (M), Filled (F), Teeth (T).

## Data Availability

The data supporting this study’s findings are available from the corresponding author upon reasonable request.

## Supplementary Materials

The following supporting information can be downloaded at: https://www.mdpi.com/article/10.3390/jcm14051616/s1, Supplementary file S1: The detailed search strategies. Supplementary Table S1: The details of Quality Assessment of the Included Studies.

## List of Abbreviations

AD	Alzheimer disease;
AMT	Abbreviated mental test;
CDR	The Clinical Dementia Rating score;
CDT	Coronal decayed teeth;
CDS	Coronal decayed surfaces;
CDFS	Coronal decayed and filled surfaces;
CFT	Coronal filled teeth;
DMFT	Decayed(D), Missing(M), Filled(F) Teeth;
DSM-IV	Diagnostic and statistical manual of mental disorders, fourth edition;
DSM-IV-TR	Diagnostic and statistical manual of mental disorders, fourth edition, text revision;
ICD-10	International Classification of Diseases, Tenth Revision;
MMSE	Mini-mental State Examination;
NINCDS-ADRDA	National Institute of Neurological and Communicative Disorders and Stroke and the Alzheimer’s Disease and Related Disorders Association;
RDS	Root decayed surfaces;
RDFS	Root decayed and filled surfaces;
shorten MMSE	A shortened version of the Mini Mental State Examination.
