# Supplementary material for: Caries Status in People with Dementia: A Systematic Review
Source: J Clin Med. 2025 Feb 27;14(5):1616. doi: 10.3390/jcm14051616 (PMC11900461; doi:10.3390/jcm14051616)
Supplement: Supplementary file 1 [file jcm-14-01616-s001.zip › Supplementary File S1 The detailed search strategies - revised.pdf]

PubMed:

Search	Query	Results
#3	#1 AND #2	1065
#2	((("dementia"[All Fields]) OR ("cognitive impairment"[All Fields])) OR ("alzheimer"[All Fields])) OR ("cognitive decline"[All Fields])	401614
#1	(((((("caries"[All Fields]) OR ("decayed teeth"[All Fields])) OR ("missing teeth"[All Fields])) OR ("filled teeth"[All Fields])) OR ("oral health"[All Fields])) OR ("oral hygiene"[All Fields])) OR ("dental health"[All Fields])	161725

WOS:

#3	#1 AND #2	2316
#2	(((TS=(dementia)) OR TS=(cognitive decline)) OR TS=(cognitive impairment)) OR TS=(alzheimer)	532694
#1	(((((((TS=(caries)) OR TS=(decayed teeth))) OR TS=(missing teeth)) OR TS=(filled teeth)) OR TS=(oral health)) OR TS=(oral hygiene)) OR TS=(dental health)	184881

Embase:

Search	Query	Results
#14	#8 AND #13	1335
#13	#9 OR #10 OR #11 OR #12	576486
#12	‘alzheimer‘	363691
#11	‘cognitive impairment’	155030
#10	‘cognitive decline’	57990
#9	‘dementia‘	312169
#8	#1 OR #2 OR #3 OR #4 OR #5 OR #6 OR #7	167643
#7	‘dental health’	26168
#6	‘oral hygiene‘	23917
#5	‘oral health‘	71195
#4	‘filled teeth‘	3492
#3	‘missing teeth‘	4492
#2	‘decayed teeth’	1201
#1	‘caries‘	81969

Scopus:

#3	#1 AND #2	647
#2	(KEY (dementia) OR KEY ("cognitive impairment") OR KEY (alzheimer) OR KEY ("cognitive decline"))	422148
#1	(KEY (caries) OR KEY ("decayed teeth") OR KEY ("missing teeth") OR KEY ("filled teeth") OR KEY ("oral health") OR KEY ("oral hygiene") OR KEY ("dental health"))	116195
