# Supplementary material for: Caries Status in People with Dementia: A Systematic Review
Source: J Clin Med. 2025 Feb 27;14(5):1616. doi: 10.3390/jcm14051616 (PMC11900461; doi:10.3390/jcm14051616)
Supplement: Supplementary file 1 [file jcm-14-01616-s001.zip › Supplementary Table S1 The details of Quality Assessment of the Included Studies.pdf]

Quality Assessment of cross-sectional studies

1. Adam H, Preston AJ. The oral health of individuals with dementia in nursing homes. Gerodontology.

Cross-sectional	Adam (2006)	Yes	No	Unclear	Not applicable
1.Were the criteria for inclusion in the sample clearly defined?	The assessments involved two populations of people aged 65 years and over, residing in four nursing homes in Cheshire, UK.	✓			
2. Were the study subjects and the setting described in detail?	The assessments involved two populations of people aged 65 years and over, residing in four nursing homes in Cheshire, UK.	✓			
3.Was the exposure measured in a valid and reliable way?	The examination firstly involved a simple assessment of the subjects' level of dementia using the Abbreviated Mental Test.	✓			
4.Were objective, standard criteria used for measurement of the condition?	Unclear.			✓	
5.Were confounding factors identified?	The two cohorts were compared for both medical history and prescription drugs taken, in attempt to limit the number of confounding factors.	✓			
6.Were strategies to deal with confounding factors stated?	The two groups were similar when compared.	✓			
7.Were the outcomes measured in a valid and reliable way?	The oral examination consisted of an assessment of: The number and position of any teeth present, using the Decayed, Missing and Filled classification.	✓			
8.Was appropriate statistical analysis used?	The Mann–Whitney U-test was used to analyze the difference between groups.	✓			

Overall appraisal: 7/8 Include ☑ Exclude □ Seek further info □

2. Syrjälä AM, Ylöstalo P, Sulkava R, Knuuttila M. Relationship between cognitive impairment and oral health: results of the Health 2000 Health Examination Survey in Finland. Acta Odontol Scand.

Cross-sectional	Syrjälä (2007)	Yes	No	Unclear	Not applicable
1.Were the criteria for inclusion in the sample clearly defined?	The present study is based on the subpopulation of those aged 55 or older.	✓			
2. Were the study subjects and the setting described in detail?	The present study is based on the subpopulation of those aged 55 or older (n=2320).	✓			
3.Was the exposure measured in a valid and reliable way?	A shortened version of the MMSE was used to measure cognitive function.	✓			
4.Were objective, standard criteria used for measurement of the condition?	The cut-off point for cognitive impairment in the shortened MMSE for this study was 12 (healthy: score 16-12), mild cognitive impairment (score 11-10) and moderate to severe impairment (9-0).	✓			
5.Were confounding factors identified?	Age, sex, education, smoking, and number of teeth.	✓			
6.Were strategies to deal with confounding factors stated?	The models were adjusted for age, sex, education, smoking, and number of teeth.	✓			
7.Were the outcomes measured in a valid and reliable way?	Dental caries was examined visually by probing on each surface of all teeth after the teeth were blown dry.	✓			
8.Was appropriate statistical analysis used?	We used Poisson's regression model to estimate the relative risk (RR) and 95% confidence intervals (CI) for continuous variables and logistic regression models to estimate the odds ratios (OR) and 95% confidence intervals for dichotomized variables.	✓			

Overall appraisal: 8/8 Include ☑ Exclude □ Seek further info □

3. Ellefsen B, Holm-Pedersen P, Morse DE, Schroll M, Andersen BB, Waldemar G. Caries prevalence in older persons with and without dementia.

Cross-sectional	Ellefsen (2008)	Yes	No	Unclear	Not applicable
1.Were the criteria for inclusion in the sample clearly defined?	Data were collected as part of an ongoing longitudinal study of elderly Caucasian people with and without newly diagnosed dementia.	✓			
2. Were the study subjects and the setting described in detail?	One hundred six dentate participants aged 66 and older were recruited from patients referred to the memory clinics at two university hospitals.	✓			
3.Was the exposure measured in a valid and reliable way?	The investigations consisted of a medical history and a physical examination in some cases. In some cases, a detailed neuropsychological examination was also performed to clarify the diagnosis.	✓			
4.Were objective, standard criteria used for measurement of the condition?	The dementia diagnosis was made according to International Classification of Diseases, Tenth Revision criteria.	✓			
5.Were confounding factors identified?	Age, sex, marital status, social relations, social position, comorbidity, functional ability, and polypharmacy	✓			
6.Were strategies to deal with confounding factors stated?	Multivariable logistic regression analysis, using backward selection, was performed to explore potential relationships while controlling for age, sex, marital status, social relations, social position, comorbidity, functional ability, and polypharmacy.	✓			
7.Were the outcomes measured in a valid and reliable way?	The clinical oral examination collected information on a wide array of clinical parameters, including dental status (teeth present, retained roots, missing teeth), caries, and so on.	✓			
8.Was appropriate statistical analysis used?	The groups with and without dementia were compared according to the various clinical oral outcome measures using bivariate analyses.	✓			

Overall appraisal: 8/8 Include ☑ Exclude □ Seek further info □

4. Hatipoglu MG, Kabay SC, Güven G. The clinical evaluation of the oral status in Alzheimer-type dementia patients.

Cross-sectional	Hatipoglu (2011)	Yes	No	Unclear	Not applicable
1.Were the criteria for inclusion in the sample clearly defined?	There were 31 patients diagnosed with AD and all were eligible to be included in the study group. Forty-seven patients of similar age and gender without known systemic disorders were assigned to the control group. The only exclusion criterion was the refusal to sign a written informed consent.	✓			
2. Were the study subjects and the setting described in detail?	All patients with AD and the controls were available from the Neurology Department of the Institution.	✓			
3.Was the exposure measured in a valid and reliable way?	Instrumental activities of daily living, index of activities of daily living scales and MMSE systems were used to evaluate the functional and cognitive functions of the participants with dementia.	✓			
4.Were objective, standard criteria used for measurement of the condition?	Those participants scoring 26 or more (out of 30) were categorized as within normal cognitive range, those scoring from 21 to 25 had mild dementia, those scoring from 11 to 20 had moderate dementia and those scoring 10 or less had severe dementia.	✓			
5.Were confounding factors identified?	Age and gender.	✓			
6.Were strategies to deal with confounding factors stated?	Forty-seven patients of similar age and gender without known systemic disorders were assigned to the control group.	✓			
7.Were the outcomes measured in a valid and reliable way?	The same dentist performed all dental examinations with the patient. The DMFT index scores were calculated and used to assess the dental health status of the participants.	✓			
8.Was appropriate statistical analysis used?	Quantitative parameters were evaluated with Student's t-test. Qualitative parameters were evaluated with Kruskal–Wallis test.	✓			

Overall appraisal: 8/8 Include ☑ Exclude □ Seek further info □

5. Hopcraft MS, Morgan MV, Satur JG, Wright FA. Edentulism and dental caries in Victorian nursing homes.

Cross-sectional	Hopcraft (2012)	Yes	No	Unclear	Not applicable
1.Were the criteria for inclusion in the sample clearly defined?	No mention.			✓	
2. Were the study subjects and the setting described in detail?	A number of 26 metropolitan Melbourne nursing homes were randomly selected using a random number generator to participate in the project.	✓			
3.Was the exposure measured in a valid and reliable way?	Patients were classified as having dementia based on their medical records.	✓			
4.Were objective, standard criteria used for measurement of the condition?	No mention.			✓	
5.Were confounding factors identified?	No mention.			✓	
6.Were strategies to deal with confounding factors stated?	No mention.			✓	
7.Were the outcomes measured in a valid and reliable way?	An experienced dental epidemiologist examined residents who consented to participate in the project. Clinical diagnostic criteria for dental caries were visually or tactilely apparent cavitation.	✓			
8.Was appropriate statistical analysis used?	No mention.			✓	

Overall appraisal: 3/8 Include ☑ Exclude ◻ Seek further info ◻

6. Philip P, Rogers C, Kruger E, Tennant M. Caries experience of institutionalized elderly and its association with dementia and functional status.

Cross-sectional	Philip (2012)	Yes	No	Unclear	Not applicable
1.Were the criteria for inclusion in the sample clearly defined?	The only inclusion criteria were that the individual must have been a resident and examined at a residential aged care facility in Perth.	✓			
2. Were the study subjects and the setting described in detail?	The study population consisted of institutionalized frail aged persons in Perth, Western Australia, and the study included data on all those participants who were examined by the dentist between January 2002 and December 2008.	✓			
3.Was the exposure measured in a valid and reliable way?	Patients were classified as having dementia based on a diagnosis of dementia provided by the patients' medical practitioner as was documented in the resident's medical records.	✓			
4.Were objective, standard criteria used for measurement of the condition?	Those persons with suspected but unconfirmed diagnosis of dementia were excluded from the study.	✓			
5.Were confounding factors identified?	No mention.			✓	
6.Were strategies to deal with confounding factors stated?	No mention.			✓	
7.Were the outcomes measured in a valid and reliable way?	Caries was assessed using both tactile and visual assessment. Caries experience was assessed using the Decayed, Missing and Filled Teeth Index (DMFT).	✓			
8.Was appropriate statistical analysis used?	Differences in outcomes were analyzed using chi-square test for categorical outcomes and student's t-test for continuous outcomes.	✓			

Overall appraisal: 6/8 Include ☑ Exclude □ Seek further info □

7. Syrjälä AM, Ylöstalo P, Ruoppi P, Komulainen K, Hartikainen S, Sulkava R, Knuuttila M. Dementia and oral health among subjects aged 75 years or older.

Cross-sectional	Syrjälä (2012)	Yes	No	Unclear	Not applicable
1.Were the criteria for inclusion in the sample clearly defined?	Elderly population aged 75 years or older.	✓			
2. Were the study subjects and the setting described in detail?	354 subjects were from the census register of the total population of people aged 75 or older living in Kuopio, in eastern Finland, on 1 November 2003 for the GeMS study.	✓			
3.Was the exposure measured in a valid and reliable way?	A history of cognitive decline was obtained by interviewing the subject and his/her relatives and examining medical records. The diagnosis was made according to the DSM-IV criteria.	✓			
4.Were objective, standard criteria used for measurement of the condition?	As stated above.	✓			
5.Were confounding factors identified?	Age, gender, education, smoking, severity of dementia and type of dwelling.	✓			
6.Were strategies to deal with confounding factors stated?	The models were adjusted for age, gender, education, smoking, severity of dementia and type of dwelling.	✓			
7.Were the outcomes measured in a valid and reliable way?	During the dental appointment two dentists (KK, PR) conducted a structured interview on oral health habits and a clinical oral examination. A dental caries diagnosis was based on a visual and tactile examination. A tooth that was both carious and filled was classified as carious, and incipient or arrested caries lesions were classified as sound.	✓			
8.Was appropriate statistical analysis used?	Risk estimates, relative risks (RR), odds ratios (OR) and 95% confidence intervals (CI) were estimated using Poisson's multivariate regression models and logistic regression models, respectively.	✓			

Overall appraisal: 8/8 Include ☑ Exclude □ Seek further info □

8. Chen X, Clark JJ, Naorungroj S. Oral health in nursing home residents with different cognitive statuses.

Cross-sectional	Chen (2013)	Yes	No	Unclear	Not applicable
1.Were the criteria for inclusion in the sample clearly defined?	(i) receiving dental care as new patients in the study clinic during the study period and (ii) residing in a NH facility – and were retrospectively selected as study participants.	✓			
2. Were the study subjects and the setting described in detail?	The study population consisted of 1626 older adults who received dental care as new patients in the study clinic during 10/1999 to 12/2006.	✓			
3.Was the exposure measured in a valid and reliable way?	A set of subjective approaches were commonly used to assess cognitive status for geriatric patients.		✓		
4.Were objective, standard criteria used for measurement of the condition?	Individuals whose medical history included International Classification of Diseases, 9th Revision, codes of 290.x, 294.1, or 331.235 or a diagnosis of Alzheimer's disease, other types of dementia, or chronic brain syndrome recorded in plain text were considered to have dementia.	✓			
5.Were confounding factors identified?	Participants' socio-demographic information (for example: age and gender) was also abstracted from dental records and included in the analysis.	✓			
6.Were strategies to deal with confounding factors stated?	Statistical results showed there were significant differences in age.		✓		
7.Were the outcomes measured in a valid and reliable way?	The existing carious conditions on dental hard tissue were verified by the radiographs of the subjects during data collection and grouped together using one variable, number of teeth being carious or retained roots.	✓			
8.Was appropriate statistical analysis used?	Poisson was developed to examine the impacts of cognitive impairment on the number of caries or retained roots and number of remaining teeth, in the study participants.	✓			

Overall appraisal: 6/8 Include ☑ Exclude ◻ Seek further info ◻

9. Campos CH, Ribeiro GR, Rodrigues Garcia RC. Oral health-related quality of life in mild Alzheimer: patient versus caregiver perceptions.

Cross-sectional	Campos (2016)	Yes	No	Unclear	Not applicable
1.Were the criteria for inclusion in the sample clearly defined?	Subjects were included in the AD patient group if they were at the mild stage of AD, were receiving treatment for AD, and wore removable prostheses. All AD subjects must be able to attend clinical session and research evaluations at Piracicaba Dental School.	✓			
2. Were the study subjects and the setting described in detail?	Participants were recruited from the PRO-CDA at Paulista State University "Julio de Mesquita Filho" (Rio Claro, São Paulo, Brazil) and from the Brazilian Alzheimer's Association (ABRAZ, Piracicaba, São Paulo, Brazil).	✓			
3.Was the exposure measured in a valid and reliable way?	AD was diagnosed by a neurologist using the International Classification of Diseases (ICD-10), Diagnostic and Statistical Manual of Mental Disorders (DSM-IV), Mini-Mental State Examination (MMSE), and Clinical Dementia Rating (CDR) scale.	✓			
4.Were objective, standard criteria used for measurement of the condition?	As stated above.	✓			
5.Were confounding factors identified?	Age, educational level, monthly income.	✓			
6.Were strategies to deal with confounding factors stated?	No mention.			✓	
7.Were the outcomes measured in a valid and reliable way?	Clinical examinations were performed by a single prosthetist dentist. The sum of the decayed, missing, and filled teeth (DMFT) was calculated as the DMFT index.	✓			
8.Was appropriate statistical analysis used?	No mention.			✓	

Overall appraisal: 6/8 Include ☑ Exclude □ Seek further info □

10. D'Alessandro G, Costi T, Alkhamis N, Bagattoni S, Sadotti A, Piana G. Oral Health Status in Alzheimer's Disease Patients: A Descriptive Study in an Italian Population.

Cross-sectional	D'Alessandro (2018)	Yes	No	Unclear	Not applicable
1.Were the criteria for inclusion in the sample clearly defined?	The selection criteria for the study sample were a confirmed diagnosis of AD and age older than 65 years.	✓			
2. Were the study subjects and the setting described in detail?	60 were institutionalized in a public elderly institute “Istituto Giovanni XXIII” in Bologna, Italy; while 60 were not institutionalized and only attended a daytime recreational center “Amarcord Cafe/A.S.P, casa Valloni” in Rimini, Italy. The data collection took place from September 2015 to January 2016.	✓			
3.Was the exposure measured in a valid and reliable way?	The medical and dental history data were collected from the medical records of the patients and/or interviewing the patients and their family members/caregivers.	✓			
4.Were objective, standard criteria used for measurement of the condition?	As stated above.	✓			
5.Were confounding factors identified?	Age.	✓			
6.Were strategies to deal with confounding factors stated?	The age of subjects did not differ between the two groups ($p = 0.940$).	✓			
7.Were the outcomes measured in a valid and reliable way?	The following clinical data were registered: dental chart, DMFT, present caries experience (DF/T index), which relates the number of carious and filled teeth to the present natural teeth; in both DMFT and DF/T, crowned teeth were considered filled teeth.	✓			
8.Was appropriate statistical analysis used?	A t-test for independent samples was used to determine differences between the control group and the total AD group. The same test was used to compare the institutionalized AD and noninstitutionalized AD subgroups.	✓			

Overall appraisal: 8/8 Include ☑ Exclude □ Seek further info □

11. Gao SS, Chen KJ, Duangthip D, Lo ECM, Chu CH. The Oral Health Status of Chinese Elderly People with and without Dementia: A Cross-Sectional Study.

Cross-sectional	Gao (2020)	Yes	No	Unclear	Not applicable
1.Were the criteria for inclusion in the sample clearly defined?	The inclusion criteria were elderly people who (1) were aged 65 years or older and (2) were cooperative to dental examination.	✓			
2. Were the study subjects and the setting described in detail?	Eight elderly daycare centers in Hong Kong were invited to join this study.	✓			
3.Was the exposure measured in a valid and reliable way?	Dementia status (yes/no) of the participants were collected from their medical record saved by the daycare centers.	✓			
4.Were objective, standard criteria used for measurement of the condition?	As stated above.	✓			
5.Were confounding factors identified?	Gender and age.	✓			
6.Were strategies to deal with confounding factors stated?	After case matching by gender and age, 129 participants with dementia were matched with 99 participants without dementia.	✓			
7.Were the outcomes measured in a valid and reliable way?	Two experienced dentists (S.S.G. and D.D.) with training in community dentistry performed the clinical examinations. They evaluated the dental caries experience of the elderly by the DMFT index according to the recommendation of the WHO. Cohen's kappa statistic was used to assess the intra- and inter-examiner agreement in clinical diagnosis.	✓			
8.Was appropriate statistical analysis used?	Independent t-tests were conducted for analyzing continuous data between dementia and nondementia groups, including the DMFT score, VPI score, and age of the participant.	✓			

Overall appraisal: 8/8 Include ☑ Exclude □ Seek further info □

12. Jockusch J, Hopfenmüller W, Nitschke I. Influence of cognitive impairment and dementia on oral health and the utilization of dental services: Findings of the Oral Health, Bite force and Dementia Study (OrBiD).

Cross-sectional	Jockusch (2021)	Yes	No	Unclear	Not applicable
1.Were the criteria for inclusion in the sample clearly defined?	Subjects were required to fulfill the following inclusion criteria: Subjects needed to be 60 years of age or older. They were included regardless of their cognitive abilities.	✓			
2. Were the study subjects and the setting described in detail?	Recruitment was randomly carried out within the patient population of a clinic specialized in gerodontology, or in cooperating facilities (long-term care facilities, geronto-psychiatric facilities).	✓			
3.Was the exposure measured in a valid and reliable way?	Subjects were stratified into five dementia evaluation groups on the basis of the Mini Mental State Examination (MMSE).	✓			
4.Were objective, standard criteria used for measurement of the condition?	Subjects were stratified into five dementia evaluation groups on the basis of the Mini Mental State Examination (MMSE)	✓			
5.Were confounding factors identified?	No mention.			✓	
6.Were strategies to deal with confounding factors stated?	No mention.			✓	
7.Were the outcomes measured in a valid and reliable way?	All clinical examinations were performed by a single investigator. The DMF/T index (D—decayed, M—missing, F— filled, T—teeth) is a measure of caries experience.	✓			
8.Was appropriate statistical analysis used?	The Kruskal–Wallis test was used to determine differences in the central tendencies of several independent samples. The Jonckheere-Terpstra test was used in the same way as the Kruskal–Wallis test but considering the priori ordering of the population in this study by means of the MMSE value grouping.	✓			

Overall appraisal: 6/8 Include ☑ Exclude □ Seek further info □

13. Auerbacher M, Gebetsberger L, Kaisarly D, Schmidmaier R, Hickel R, Drey M. Oral health in patients with neurodegenerative and cerebrovascular disease: a retrospective study.

Cross-sectional	Auerbacher (2022)	Yes	No	Unclear	Not applicable
1.Were the criteria for inclusion in the sample clearly defined?	Based on medical diagnosis, for this study, all adults with MS, PD, D, and CVD with a degree of disability and/or level of care were selected.	✓			
2. Were the study subjects and the setting described in detail?	Data from 152 patients with neurodegenerative or CVDs and 30 controls were extracted from the patient files and evaluated. All patients received dental treatment from the same examiner in the division for people with special needs at the university hospital between March 2012 and October 2020.	✓			
3.Was the exposure measured in a valid and reliable way?	Based on medical diagnosis, for this study, all adults with MS, PD, D, and CVD with a degree of disability and/or level of care were selected.	✓			
4.Were objective, standard criteria used for measurement of the condition?	As stated above.	✓			
5.Were confounding factors identified?	Age and sex.	✓			
6.Were strategies to deal with confounding factors stated?	Group differences were analyzed by multiple linear and logistic regression analysis for several dental outcomes adjusted for sex and age.	✓			
7.Were the outcomes measured in a valid and reliable way?	All patients received dental treatment from the same examiner in the division for people with special needs at the university hospital between March 2012 and October 2020. The dental status, number of decayed, missing, and filled teeth were recorded on the first visit.	✓			
8.Was appropriate statistical analysis used?	Significant differences between the groups for quantitative variables were calculated using Student’s t-test and one-way ANOVA. Significant differences between the groups for categorical variables were calculated using the chi-square test and Fisher’s exact test.	✓			

Overall appraisal: 8/8 Include ☑ Exclude □ Seek further info □

Quality Assessment of case–control studies

1. Ship JA. Oral health of patients with Alzheimer's disease.

Case–control study	Ship (1992)	
**Selection**<br>1) Is the case definition adequate<br>a) yes, with independent validation*<br>b) yes, e.g., record linkage or based on self-reports<br>c) no description	A diagnosis of definite, probable and possible Alzheimer's was made according to NINCDS-ADRDA criteria, after patients were screened vigorously to exclude other medical, neurological or psychiatric conditions.	a*
2) Representativeness of the cases<br>a) consecutive or obviously representative series of cases*<br>b) potential for selection biases or not stated	Forty-one community-dwelling whites with a clinical diagnosis of Alzheimer's disease were evaluated as part of an ongoing longitudinal study at the National Institute on Aging (NIA) at the Clinical Center of the National Institutes of Health.	a*
3) Selection of Controls<br>a) community controls*<br>b) hospital controls<br>c) no description	Forty-nine healthy white people were selected as controls. All were community dwelling individuals who underwent rigorous medical, neurological and laboratory screening.	a*
4) Definition of Controls<br>a) no history of disease (end point)*<br>b) no description of source	All were community-dwelling individuals who underwent rigorous medical, neurological and laboratory screening. Control individuals were neither being treated for any systemic disease nor taking prescription medications.	a*

**Comparability**<br>5) Comparability of cases and controls on the basis of the design or analysis<br>a) study controls for age *<br>b) study controls for any additional factor*	As no gender differences were observed for any of the clinical parameters, analyses were performed with men and women combined.	b*
**Exposure**<br>6) Ascertainment of exposure<br>a) secure record (e.g., surgical records) *<br>b) structured interview where blind to case/control status*<br>c) interview not blinded to case/control status<br>d) written self-report or medical record only<br>e) no description	The diagnosis of each patient was unknown at the time of the clinical examination.<br>The number of teeth (excluding third molars), decayed-missing-filled-teeth score, and the number of teeth with coronal and cervical caries and restorations were recorded.	a*
7) Same method of ascertainment for cases and controls<br>a) yes*<br>b) no	As stated above.	a*
8) Non-Response rate<br>a) same rate for both groups *<br>b) non respondents described<br>c) rate different and no designation secure record (e.g., surgical records)	No mention	b
Risk of bias		moderate
Final quality assessment		moderate
* point		7/9

2. Jones JA, Lavallee N, Alman J, Sinclair C, Garcia RI. Caries incidence in patients with dementia.

**Case–control study**	Jones (1993)	
**Selection**<br>1) Is the case definition adequate<br>a) yes, with independent validation*<br>b) yes, e.g., record linkage or based on self-reports<br>c) no description	Persons with a diagnosis of possible or probable dementia of the Alzheimer's type are typically followed at three-month intervals from the time of diagnosis through long-term care, death, and post-mortem.	a*
2) Representativeness of the cases<br>a) consecutive or obviously representative series of cases*<br>b) potential for selection biases or not stated	45 patients of the Longitudinal Study of Dementia were recruited for this study via letters to their next of kin and the subsequent procurement of their informed consent.	b
3) Selection of Controls<br>a) community controls*<br>b) hospital controls<br>c) no description	Similar data of the controls were then retrospectively abstracted from the Department of Veterans Affairs (VA) Dental Longitudinal Study (DLS).	b
4) Definition of Controls<br>a) no history of disease (end point)*<br>b) no description of source	Free of dementia. (detail shown in reference [15])	a*
**Comparability**<br>5) Comparability of cases and controls on the basis of the design or analysis<br>a) study controls for age *<br>b) study controls for any additional factor*	Two comparison subjects per each DAT subject were selected from the VA Dental Longitudinal Study, matching for age, number of teeth, and education.	a*<br>b*
**Exposure**<br>6) Ascertainment of exposure<br>a) secure record (e.g., surgical records) *	The examiner for the DAT participants used the scoring forms and protocol established by the National Institute of Dental Research (NIDR) and used in their 1985-86 survey of employed	a*

b) structured interview where blind to case/control status*<br>c) interview not blinded to case/control status<br>d) written self-report or medical record only<br>e) no description	adults and seniors. Criteria for coronal caries in the DLS group are identical to the NIDR criteria.	
7) Same method of ascertainment for cases and controls<br>a) yes*<br>b) no	As stated above.	a*
8) Non-Response rate<br>a) same rate for both groups *<br>b) non respondents described<br>c) rate different and no designation secure record (e.g., surgical records)	Of the original 34 individuals entered into the study, 9 died, 1 became edentulous, and 1 moved.	c
Risk of bias		Moderate
Final quality assessment		Moderate
* point		6/9

3. Bramanti E, Bramanti A, Matacena G, Bramanti P, Rizzi A, Cicciù M. Clinical evaluation of the oral health status in vascular-type dementia patients. A case–control study.

**Case–control study**	Bramanti (2015)	
**Selection**<br>1) Is the case definition adequate<br>a) yes, with independent validation*<br>b) yes, e.g., record linkage or based on self-reports<br>c) no description	A number of 86 consecutive subjects staying at IRCSS Neurolesi "Bonino-Pulejo" in Messina were diagnosed by medical specialist as having vascular dementia.	a*
2) Representativeness of the cases<br>a) consecutive or obviously representative series of cases*<br>b) potential for selection biases or not stated	As stated above.	b
3) Selection of Controls<br>a) community controls*<br>b) hospital controls<br>c) no description	No mention.	c
4) Definition of Controls<br>a) no history of disease (end point)*<br>b) no description of source	MMSE≥26	a*
**Comparability**<br>5) Comparability of cases and controls on the basis of the design or analysis<br>a) study controls for age *<br>b) study controls for any additional factor*	The dementia patients and the controls were age-/sex-matched.	a*<br>b*
**Exposure**<br>6) Ascertainment of exposure<br>a) secure record (e.g., surgical records) *<br>b) structured interview where blind to case/control status*	Caries was evaluated by using tactile, visual and mirror assessment and measured by decaying, missing and filled teeth (DMFT) index.	a*

c) interview not blinded to case/control status<br>d) written self-report or medical record only<br>e) no description		
7) Same method of ascertainment for cases and controls<br>a) yes*<br>b) no	As stated above.	a*
8) Non-Response rate<br>a) same rate for both groups *<br>b) non respondents described<br>c) rate different and no designation secure record (e.g., surgical records)	No mention.	b
Risk of bias		Moderate
Final quality assessment		Moderate
* point		6/9

4. Chu CH, Ng A, Chau AM, Lo EC. Oral health status of elderly Chinese with dementia in Hong Kong.

**Case–control study**	Chu (2015)	
**Selection**<br>1) Is the case definition adequate<br>a) yes, with independent validation*<br>b) yes, e.g., record linkage or based on self-reports<br>c) no description	The target population selected for this project consisted of elderly people who a) were ≥ 60 years of age, b) had been diagnosed with dementia and c) were fit for periodontal assessment with probing.	a*
2) Representativeness of the cases<br>a) consecutive or obviously representative series of cases*<br>b) potential for selection biases or not stated	Recruitment of participants was carried out in the day-care centers of the Hong Kong Alzheimer's Disease Association and St. James' Settlement Kin Chi Dementia Care Support Service Center in March 2010.	b
3) Selection of Controls<br>a) community controls*<br>b) hospital controls<br>c) no description	After the sample size estimation, a quota sample of 82 elderly people was invited to the study through the daycare centers via email and letters of invitation.	b
4) Definition of Controls<br>a) no history of disease (end point)*<br>b) no description of source	A sex- and age-matched group of 59 healthy elderly people without dementia were recruited and examined as the control group.	a*
**Comparability**<br>5) Comparability of cases and controls on the basis of the design or analysis<br>a) study controls for age *<br>b) study controls for any additional factor*	As stated above.	a*<br>b*
**Exposure**<br>6) Ascertainment of exposure<br>a) secure record (e.g., surgical records) *	Clinical examination was conducted by two calibrated, trained examiners using dental mirrors with LED lights and WHO probes. The oral examination was performed according to the	a*

b) structured interview where blind to case/control status*<br>c) interview not blinded to case/control status<br>d) written self-report or medical record only<br>e) no description	guidelines of the World Health Organization (1997).<br>The DMFT index was used to measure the caries experience of permanent teeth.	
7) Same method of ascertainment for cases and controls<br>a) yes*<br>b) no	As stated above.	a*
8) Non-Response rate<br>a) same rate for both groups *<br>b) non respondents described<br>c) rate different and no designation secure record (e.g., surgical records)	The response rate of the dementia group was 72%.	c
Risk of bias		moderate
Final quality assessment		moderate
* point		6/9

5. Cestari JA, Fabri GM, Kalil J, Nitrini R, Jacob-Filho W, de Siqueira JT, Siqueira SR. Oral Infections and Cytokine Levels in Patients with Alzheimer's Disease and Mild Cognitive Impairment Compared with Controls.

**Case–control study**	Cestari (2016)	
**Selection**<br>1) Is the case definition adequate<br>a) yes, with independent validation*<br>b) yes, e.g., record linkage or based on self-reports<br>c) no description	Twenty-five patients with AD diagnosed according to the criteria of the National Institute for Communicative Disorders and Stroke (Alzheimer's Disease and Related Disorders Association) (NINCDS-ADRDA) were evaluated.	a*
2) Representativeness of the cases<br>a) consecutive or obviously representative series of cases*<br>b) potential for selection biases or not stated	All patients were diagnosed by an experienced neurologist of the Group of Behavior and Cognitive Neurology of the Hospital das Clinicas (Medical School of the University of Sao Paulo).	b
3) Selection of Controls<br>a) community controls*<br>b) hospital controls<br>c) no description	They were non-demented elderly that had been followed at the Group of Multidisciplinary Assistance of Elderly of the Geriatric Clinic at the same hospital.	b
4) Definition of Controls<br>a) no history of disease (end point)*<br>b) no description of source	They were non-demented elderly that had been followed at the Group of Multidisciplinary Assistance of Elderly of the Geriatric Clinic at the same hospital.	a*
**Comparability**<br>5) Comparability of cases and controls on the basis of the design or analysis<br>a) study controls for age *<br>b) study controls for any additional factor*	There were no differences in age, gender, marital status, educational status and economic status between two groups.	a*<br>b*
**Exposure**<br>6) Ascertainment of exposure<br>a) secure record (e.g., surgical records) *	A single trained dentist performed the complete oral exam. Caries was measured by the decayed, missing, and filled tooth index	a*

b) structured interview where blind to case/control status*<br>c) interview not blinded to case/control status<br>d) written self-report or medical record only<br>e) no description		
7) Same method of ascertainment for cases and controls<br>a) yes*<br>b) no	As stated above.	a*
8) Non-Response rate<br>a) same rate for both groups *<br>b) non respondents described<br>c) rate different and no designation secure record (e.g., surgical records)	No mention.	b
Risk of bias		moderate
Final quality assessment		moderate
* point		6/9

6. Aragón F, Zea-Sevilla MA, Montero J, Sancho P, Corral R, Tejedor C, Frades-Payo B, Paredes-Gallardo V, Albaladejo A. Oral health in Alzheimer's disease: a multicenter case–control study.

**Case–control study**	Aragón (2018)	
**Selection**<br>1) Is the case definition adequate<br>a) yes, with independent validation*<br>b) yes, e.g., record linkage or based on self-reports<br>c) no description	All of the participants of the Alzheimer group fulfilled the criteria for dementia caused by Alzheimer's disease based on McKhann et al. diagnosed criteria, regardless of their stage.	a*
2) Representativeness of the cases<br>a) consecutive or obviously representative series of cases*<br>b) potential for selection biases or not stated	70 were individuals suffering from AD and residents at the Alzheimer Center Reina Sofia Foundation (Madrid, Spain) and at the Alzheimer State Reference Center (and other dementias) Salamanca (Spain).	b
3) Selection of Controls<br>a) community controls*<br>b) hospital controls<br>c) no description	Thirty-six of the participants formed the healthy control group and were selected from among the patients' caregivers (family members or friends having the same sociocultural level and from the same reference center, although mostly of them were younger than the patients).	b
4) Definition of Controls<br>a) no history of disease (end point)*<br>b) no description of source	The control group was healthy people who had no neurological disease and who were able to collaborate properly.	a*
**Comparability**<br>5) Comparability of cases and controls on the basis of the design or analysis<br>a) study controls for age *<br>b) study controls for any additional factor*	The average age of the Alzheimer's patients was significantly higher than that of the controls.<br>Both controls and cases had the same sociocultural level.	b*
**Exposure**<br>6) Ascertainment of exposure	All of the participants were later subjected to an oral, dental, and periodontal examination, according to the criteria established by	a*

a) secure record (e.g., surgical records) *<br>b) structured interview where blind to case/control status*<br>c) interview not blinded to case/control status<br>d) written self-report or medical record only<br>e) no description	the World Health Organization, to check for caries in teeth (DMFT index: sum of decayed, missed and filled teeth) and in surfaces.	
7) Same method of ascertainment for cases and controls<br>a) yes*<br>b) no	As stated above.	a*
8) Non-Response rate<br>a) same rate for both groups *<br>b) non respondents described<br>c) rate different and no designation secure record (e.g., surgical records)	No mention.	b
Risk of bias		moderate
Final quality assessment		moderate
* point		5/9

7. Panzarella V, Mauceri R, Baschi R, Maniscalco L, Campisi G, Monastero R. Oral Health Status in Subjects with Amnestic Mild Cognitive Impairment and Alzheimer's Disease: Data from the Zabút Aging Project.

**Case–control study**	Panzarella（2022）	
**Selection**<br>1) Is the case definition adequate<br>a) yes, with independent validation*<br>b) yes, e.g., record linkage or based on self-reports<br>c) no description	The diagnosis of dementia was ascertained by specialists according to the DSM-IV-TR criteria and probable AD was diagnosed according to the National Institute on Aging and the Alzheimer's Association criteria.	a*
2) Representativeness of the cases<br>a) consecutive or obviously representative series of cases*<br>b) potential for selection biases or not stated	Participants were recruited during the 10-year follow-up of the Zab´ut Aging Project (ZAP), a population-based cohort study conducted in a rural community with a low educational level in the province of Agrigento (Sicily, Italy).	a*
3) Selection of Controls<br>a) community controls*<br>b) hospital controls<br>c) no description	As stated above.	a*
4) Definition of Controls<br>a) no history of disease (end point)*<br>b) no description of source	20 cognitively normal individuals without cognitive and functional impairment [CONS].	a*
**Comparability**<br>5) Comparability of cases and controls on the basis of the design or analysis<br>a) study controls for age *<br>b) study controls for any additional factor*	No significant differences in age between the groups.<br>The overall sample had a mean year of educational level of 4.81±3.01, without significant difference between the groups.	a*<br>b*
**Exposure**<br>6) Ascertainment of exposure	The initial data collection regarded an evaluation of dental status according to the Decayed, Missing, and Filled Teeth (DMFT)	a*

a) secure record (e.g., surgical records) *<br>b) structured interview where blind to case/control status*<br>c) interview not blinded to case/control status<br>d) written self-report or medical record only<br>e) no description	index.	
7) Same method of ascertainment for cases and controls<br>a) yes*<br>b) no	All participants (n = 60) underwent a complete assessment of oral health status.	a*
8) Non-Response rate<br>a) same rate for both groups *<br>b) non respondents described<br>c) rate different and no designation secure record (e.g., surgical records)	No mention.	b
Risk of bias		low
Final quality assessment		high
* point		8/9
